# Alternative CAR Therapies: Recent Approaches in Engineering Chimeric Antigen Receptor Immune Cells to Combat Cancer

**DOI:** 10.3390/biomedicines10071493

**Published:** 2022-06-24

**Authors:** Carlos Moreno, Christopher Haynie, Abigail Cheever, K. Scott Weber

**Affiliations:** Department of Microbiology and Molecular Biology, Brigham Young University, Provo, UT 84602, USA; carlosmoreno943@gmail.com (C.M.); christopher.j.haynie7@gmail.com (C.H.); abigail.kay.johnson@gmail.com (A.C.)

**Keywords:** T cell, chimeric antigen receptor, CAR, NK cell, macrophage, autoimmune, cancer, immunotherapy, autoreactive B cell, cytotoxicity

## Abstract

For nearly three decades, chimeric antigen receptors (CARs) have captivated the interest of researchers seeking to find novel immunotherapies to treat cancer. CARs were first designed to work with T cells, and the first CAR T cell therapy was approved to treat B cell lymphoma in 2017. Recent advancements in CAR technology have led to the development of modified CARs, including multi-specific CARs and logic gated CARs. Other immune cell types, including natural killer (NK) cells and macrophages, have also been engineered to express CARs to treat cancer. Additionally, CAR technology has been adapted in novel approaches to treating autoimmune disease and other conditions and diseases. In this article, we review these recent advancements in alternative CAR therapies and design, as well as their mechanisms of action, challenges in application, and potential future directions.

## 1. Introduction

### 1.1. Development of CAR T Cells

Recent approaches to cancer treatment have focused on immunotherapy options, due to their potential to improve both patient survival and quality of life compared to previous standards of care [1]. Cancer immunotherapy harnesses the power of the immune system to prevent, control, or eliminate cancer. One approach to enhancing the immune response in cancer immunotherapy is to direct immune cell effector functions toward cancer cells. A major challenge with directing the immune response against cancer is the difficulty immune cells have in recognizing and discriminating healthy and cancer cells [2]. Cancer cells can be identified by tumor associated antigens (TAAs), proteins that have elevated levels in cancer, or can be identified by tumor specific antigens (TSAs), proteins only present in cancer cells. TAAs and TSAs can be difficult to identify because cancer cells often only have a few mutations and do not manifest discernible differences on the cell surface when compared with healthy cells.

In addition to the difficulty of targeting cancer cells in solid tumors, cancer cells rapidly consume surrounding nutrients and alter local cytokine production, creating a nutrient scarce and immunosuppressive tumor microenvironment (TME) that can promote the exhaustion of infiltrating and surrounding immune cells [3]. Tumor cells themselves release anti-inflammatory cytokines that alter the functional phenotypes of infiltrating immune cells and even support tumor growth [4]. Some cell death occurs during tumor development, which signals to recruit first-responders, such as neutrophils and macrophages [5]. However, since the cancer is derived from self cells, cancer cells may not generate a pro-inflammatory response, and the first responding cells may switch instead to an immunoregulatory phenotype, protecting or even promoting tumor growth. The anti-tumor functions of natural killer (NK) cells and cytotoxic T cells may then be suppressed by cytokine signaling from both the tumor and tumor associated macrophages (TAM). These immunosuppressive signals can increase rates of cell anergy, which is a lack of cellular activation following antigen encounter, where the lymphocyte remains alive for extended periods of time but in a hyporesponsive state [3,6]. The net result is immunosuppression that inhibits the anti-tumor activities of the immune system. To counteract these effects, cancer immunotherapies are designed to enable immune cells to resist the immunosuppressive properties and nutrient scarcity of the TME or alter the TME to allow immune cells to have an effective response against the cancer [7,8,9,10]. With proper stimulation, the phenotype of immune cells in the TME can be returned to the pro-inflammatory state and allow for the activation and immune targeting of the cancer [11].

T cells are adaptive immune cells that are critical for removing both cells infected with intracellular pathogens and cancer cells. To become fully activated, T cells must receive primary stimulation through the interaction of the T cell receptor (TCR) and specific antigen peptide presented on the major histocompatibility complex (MHC). In addition, T cells must receive secondary co-stimulation from co-receptors and cytokine signaling to fully activate and differentiate. As principal components of an adaptive immune response, T cells play an important role in combating and destroying cancer cells. However, T cells must overcome the evasion mechanisms employed by cancer cells. Cancer cells employ multiple strategies to evade or disrupt T cell function, including downregulation of normal peptide-MHC presentation to avoid T cell detection, and immune cell suppression signaling via anti-inflammatory cytokine secretion and inhibitory co-receptor signaling resulting in T cell exhaustion [12]. Chimeric antigen receptor (CAR) T cells circumvent several limitations in treating cancer by combining antibody specificity and T cell cytotoxicity to directly target and destroy cancer cells.

CARs were first developed in 1987 by Kuwana et al. as a conceptual comparison between T and B cell receptor variable regions [13]. CAR T cells were first proposed as a model for immunotherapy in 1989 by Dr. Zelig Eshhar [14]. However, these initial CAR T cells were not persistent and lacked necessary co-stimulation to remain active and eliminate cancer cells. In 1998, Dr. Michel Sadelain and colleagues showed that the efficacy of CAR T cells significantly increased with the addition of the co-stimulatory domain of CD28 [15]. With this breakthrough, Dr. Sadelain and colleagues developed the first functional CAR T cells in 2002 and then showed that CAR T cells could kill leukemia cells in a mouse model [16,17]. Next-generation CAR T cells have been developed to address problems with persistence and are now viable treatment options. Phase 1 clinical trials using CD19 specific CARs to treat leukemia began in 2010 and led to the FDA-approval of CD19 CAR therapy to treat B cell malignancies in 2017. In 2022, Dr. Carl June and colleagues found that CAR T cells persisted in two patients in remission that had been infused with CAR T cells over 10 years earlier [18,19].

### 1.2. Generations of CAR T Cells

**Figure 1:**
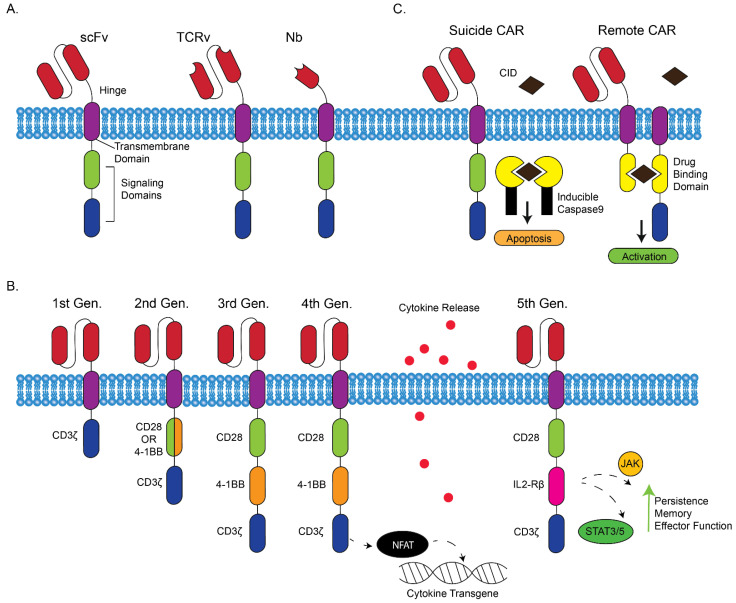
**Overview of CAR constructs, the five CAR generations, and regulatory options.** (**A**) Various CAR constructs are shown with scFv, TCR variable region (TCRv), and nanobody (Nb) extracellular domains. (**B**) CAR generation constructs are shown with the various changes to internal signaling domains. First generation CARs only include one intracellular signaling domain (CD3ζ). Second generation CARs include an additional co-stimulatory domain of CD28, 4-1BB, or OX-40. Third generation CARs include two co-stimulatory domains. Fourth generation CARs include a cytokine transgene initiated through NFAT signaling. Fifth generation CARs include IL-2Rβ to initiate JAK/STAT signaling. (**C**). Suicide CARs can be stopped, and remote CARs can be initiated, by a chemical inducer of dimerization (CID).

The basic structure of a CAR includes an extracellular target-binding domain, transmembrane domains, and intracellular signaling domains (Figure 1A).

The extracellular target-binding region is composed of a single chain fragment variable (scFv) from antibody light and heavy chain variable regions. While scFvs are the most common extracellular domain, some CAR designs include a high-affinity TCR to detect intracellular TAAs or include a nanobody domain derived from camelid antibodies (Figure 1A) [20,21]. This extracellular target-binding domain is joined by the transmembrane domain to various signaling domains consisting of a primary signaling domain (CD3ζ) and co-stimulatory domains (CD28 or 4-1BB or OX-40) [22,23,24]. First generation CAR T cells only included CD3ζ as their internal signaling domain; however, they were not effective because the cells lacked co-stimulation to become fully activated. Further generations of CARs helped to resolve activation and persistence difficulties by adjusting signaling and cellular patterns to better match the natural immune activation signal (Figure 1B). Second generation CARs have an additional costimulatory domain (e.g., CD28 or 4-1BB or OX-40), which provides proper co-stimulation that is necessary for full T cell activation. Third generation CARs sought to improve upon the second generation by adding an additional co-stimulatory domain as part of the primary signal, such as a combination of both CD28 and 4-1BB, or OX40 in conjunction with CD3ζ [25]. Fourth generation CAR T cells, also known as T cells redirected for antigen-unrestricted cytokine-initiated killing (TRUCKs), include a transgenic cytokine, such as IL-12, which encodes a secreted cytokine that is induced through NFAT signaling. Cytokines secreted by TRUCKs (IL-12, IL-7, IL-15, IL-18 etc.) help to support tumor destruction and are localized in the tissue targeted by the CAR T cell [26]. While previous generations of CARs sought to increase effector function of T cells, fifth generation CARs seek to address the potential adverse effects that can be present in CAR T cell treatment, including neurotoxicity and cytokine release syndrome (CRS). CRS is a sudden and widespread release of inflammatory cytokines by T cells composed predominantly of IL-6, INF-γ, and IL-10. The purpose of fifth generation CAR T cells is to localize cytokine signaling only in the presence of the cancer antigen. Fifth generation CAR T cells are the newest proposed mode of action that include an intracellular signaling domain for cytokine signaling receptors, such as IL2-Rβ, which will trigger the JAK/STAT pathway to stimulate effector function, persistence, and memory, and provide stimulation that would come through normal cytokine signaling. Cytokine production and stimulation induced through the JAK/STAT pathway was shown to promote a similar response in the cell as if it had received IL-21 stimulation. The net effect is local effector cytokine production and stimulation, only when in direct contact with the tumor antigen, to mitigate overabundant cytokine production that leads to CRS [27,28]. Alternative CAR T cell formats include remote CAR T cells that have inducible activation responses, and suicide CARs that include a suicide signal once treatment is concluded or no longer needed (Figure 1C) [29,30].

Interest surrounding CAR T cell therapies has increased over the past decade, particularly for lymphomas and leukemias [31,32,33,34,35]. Despite the current success in CAR T cell treatment approaches for leukemias and lymphomas, CAR T cells have shown limited effectiveness in solid tumor cancers [36,37]. One of the major difficulties and drawbacks to the potential of CAR T cell therapies in solid tumors is in the TME. Competition for resources, immunosuppressive stimuli provided by tumor cells and immunoregulatory immune cells, and stromal extracellular matrix (ECM) of solid tumors can dramatically decrease the effectiveness of CAR T cells to be able to infiltrate tumors and eliminate tumor cells [3,38]. In addition to the TME, one challenge to adoptive CAR T cell immunotherapies is T cell source availability for treatment. CAR T cell construction is limited to patient T cells because donor T cells must resolve graft versus host disease (GvHD) or have human leukocyte antigen (HLA) matching. CAR T cell development from donor sources requires adjustments in surface protein expression to avoid rejection by the new host [39]. Another problem with patient-derived T cells is the potential of T cell exhaustion, due to previous treatment stressing the immune cells.

Additionally, many clinical trials and current treatments target markers present on cancer cells and on healthy tissue. CAR T cell engineering requires at least one TSA or TAA to be able to effectively target cancerous cells. Because TAAs can be present on healthy cells as well as cancerous cells, CAR T cells can have off-target cytotoxic effects. Current CAR T cell therapies against CD19 have reported debilitating effects on the humoral immune system post-treatment, due to the elimination of healthy B cells that also express CD19 [40]. Another concern with CAR T cells is the potential of adverse effects including CRS and neurotoxicity, although efforts have been made to limit these effects [41]. CRS is one of the biggest drawbacks of CAR T cell therapy and can lead to rapid death [42,43]. Although CRS is a concern when using a CAR T cell therapy, when carefully monitored, the effects can be treated to prevent patient death [44]. Remote CARs seek to address CRS by controlling T cell activation, while suicide CARs include a kill switch to effectively shut down the CAR T cells to stop the overabundance of inflammation (Figure 1C). Currently, there are over 500 CAR T cell therapies in clinical trials worldwide and 6 CAR T cell therapies approved for full use by the FDA [25,45,46].

### 1.3. Six FDA Approved CAR Cancer Immunotherapies

Of the six CAR T cell therapies currently FDA approved, all are directed for use in blood cancers. Four specifically target CD19 in B cell lymphomas [25,47,48]. CD19 is a B cell marker present on all mature B cells. In B cell lymphomas, CD19 can be utilized as a cancer biomarker, as it is consistently present in B cell lymphomas [49]. Since most B cell malignancies express CD19 at high levels, it is commonly used as a diagnostic tool and target for blood cancers, such as Burkett’s B cell lymphoma, B cell acute lymphoblastic leukemia (B-ALL), and B cell non-Hodgkin lymphoma [32,33,47,50,51]. There is a success rate of more than 80% complete remission among B cell lymphoma patients treated with CAR T cell therapy [52,53]. CAR T cell therapy has provided a unique and viable avenue of treatment for B cell lymphoma patients where previously there was no treatment option available. The remaining two therapies target B-cell maturation antigen (BCMA). BCMA is a member of the tumor necrosis factor receptor family and is expressed at normal levels on the surface of mature B cells and becomes overexpressed in multiple myeloma [54]. The first BCMA CAR T cell therapy was approved in 2021, and in February 2022, the second was approved for the treatment of patients with relapsed multiple myeloma [25,48,55]. The most recent FDA-approved CAR T cell therapy, ciltacabtagene autoleucel, was reported to have an overall response rate of 97% and a 67% complete response rate [56]. Despite only six CAR T cell therapies being fully FDA approved, there are over five hundred more therapies currently in clinical trial. Many of these CAR T cell therapies in clinical trial are oriented towards common neoantigens, such as epidermal growth factor receptor (EGFR) or human epidermal growth factor receptor 2 (HER2). Research discovering novel neoantigens is ongoing, as this is key in developing CARs.

## 2. Multi-Specific CAR T Cells

### 2.1. Addressing the Challenge of Antigen Escape Using Car Technology

**Figure 2:**
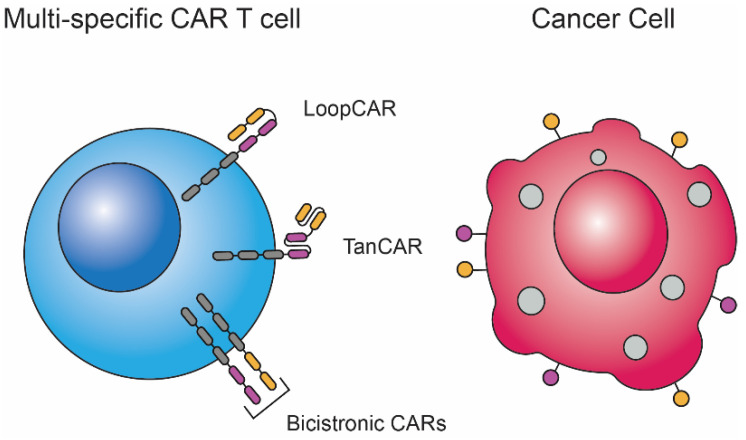
**Multi-specific CAR T cell designs.** Currently, there are at least three approaches to designing multi-specific CAR T cells that recognize more than one antigen. The most basic design for engineering multi-specific CAR T cells is transducing two (bicistronic) or more CAR constructs into the same cell, which allows multiple antigens to be targeted. Tandem CARs (TanCAR) are CAR constructs with more than one scFv binding domain joined in tandem, allowing multi-specificity. Tandem CAR constructs containing a loop structure (LoopCAR) are also a recent design, which in some cases may be more efficient at bivalent binding compared to TanCARs, as described by Qin et al. [61].

Thus far, we have described monovalent CAR T cell therapies that target only one antigen. These monovalent therapies face the challenge of antigen escape (loss of the target antigen on tumor cells due to downregulation or mutations), as well as the heterogeneity of antigen expression on tumor cells between patients [57]. Antigen loss can lead to relapses and prevent remission in patients [58]. In fact, 60% of relapses during anti-CD19 CAR T cell therapies to treat relapsing B-ALL occur due to antigen loss, and up to one-third of patients may experience relapse due to CD19 antigen loss [59]. One approach to addressing this challenge is introducing two different CARs into the same T cell, and this can be carried out using bicistronic CAR, TanCAR, or LoopCAR methods (Figure 2) [57,59,60,61]. In 2016, Ruella et al. genetically modified T cells to express both anti-CD19 and anti-CD123 CARs [59]. They observed that bicistronic CD19/123 CAR T cells exhibited enhanced early (day 6) and long-term anti-leukemia effects when treating a primary B-ALL mouse model compared to anti-CD19 and anti-CD123 CAR T cell treatments, either individually or combined [59]. A similar approach to preventing relapses due to antigen loss is developing multivalent CARs, which contain multiple binding domains in the same CAR construct.

### 2.2. Development of TanCARs and LoopCARs

In 2013, Grada et al. introduced a novel bispecific CAR into T cells using an SFG retroviral vector [62]. Since the scFv regions of the CAR were bound in tandem by a linker, they termed the construct “TanCAR” (Figure 2). The authors’ aim was to counteract antigen escape and to enable the simultaneous targeting of antigens presented on tumor cells and components of the TME. In their study, anti-CD19/HER2 TanCAR T cells were able to specifically target HER2-expressing Daoy cells and CD19-expressing Raji cells in vitro. Importantly, they observed enhanced anti-tumor activity by adoptively transferred TanCAR T cells in a Daoy.TET.CD19 xenograft SCID mouse model expressing HER2 and CD19 induced by doxycycline administration. Enhanced anti-tumor activity occurred when the TanCAR T cells recognized simultaneously both HER2 and CD19 within the tumor (median survival >60 days with 50% of mice surviving >80 days), compared to TanCAR T cells only recognizing HER2 in HER2+/CD19- tumors (median survival of 44 days). These results may indicate the potential advantage of targeting multiple cancer antigens to enhance the anti-tumor activity of CAR T cells. Although, differences in the activation of TanCAR T cells recognizing one or both antigens may be a result of altered binding affinity and steric hinderance, due to the tandem binding of scFv regions.

Since the study of Grada et al., other studies have focused on developing multi-specific CAR therapies while optimizing CAR design to maximize multiple antigen recognition and the anti-tumor activity of transduced cells [57,61,63]. In 2018, Qin et al. sought to resolve the challenge of relapsing B-ALL due to CD19 antigen loss [57]. To do so, they engineered T cells expressing bivalent anti-CD19/CD22 TanCARs. Their study demonstrates the need for the testing and optimization of multi-valent CAR constructs, as the authors developed four TanCARs with varying V_H_ and V_L_ linker lengths, linker lengths between the two scFv regions, and scFv orders. These four TanCARs varied in their surface expression, antigen recognition, and tumor clearance in vitro. The authors even engineered CARs that incorporated loop structures with their scFv regions called “LoopCARs” (Figure 2). T cells expressing the sixth iteration of their bivalent LoopCARs were effective at binding and eliminating CD19+/CD22+ and CD19- patient-derived xenograft tumor cells.

Currently, there are no FDA-approved bivalent CAR T cell therapies. However, recent success in developing multi-specific CAR T cell therapies to prevent tumor escape via antigen loss and enhance cancer cell clearance in vitro and in mouse models has led to several therapies entering clinical trials. These trials have been completed or are being conducted to test the safety and efficacy of bivalent CAR T cells against several cancer types, including multiple myeloma (NCT04662099), B-ALL (NCT03233854, NCT03289455, NCT03919526, NCT04499573), diffuse large B cell lymphoma (LBCL) (NCT03287817, NCT04703686), Non-Hodgkin lymphoma (NCT04703686), and mantle cell lymphoma (NCT04703686). Adverse effects, such as CRS and neurotoxicity, are still concerns during bivalent CAR T cell therapies. This is demonstrated by a completed phase 1 trial investigating the manufacturing feasibility and safety of CD19/CD22 bivalent CAR T cell therapy to treat B-ALL and LBCL (NCT03233854). The investigators in this study reported that 29 out of 38 patients (76%) who received the bivalent CARs experienced some level of CRS, with 2 patients experiencing grade ≥3 CRS [64]. A total of 14 patients experienced some level of neurotoxicity, with 4 experiencing grade ≥ 3 neurotoxicity. However, all episodes of CRS and neurotoxicity were resolved. Another phase I clinical trial conducted by Shalabi et al. reported that 10 out of 20 patients treated with CD19/CD22 bivalent CAR T cells to treat B-ALL in children and adults experienced CRS, with 3 having grade 3 CRS [65]. They also report only one patient experiencing neurotoxicity, though it was grade 3.

On-target, off tumor effects during CAR T cell therapy have been reported, a phenomenon characterized by CAR T cells recognizing their specific antigen on healthy, non-cancerous cells [66]. This may occur due to antigen expression profiles being similar between cancerous and healthy cells. Intuitively, targeting multiple antigens simultaneously by CAR T cells may raise the chances of off-target toxicity, but the extent of multi-specific CAR T cells exhibiting increased off-target effects has not been extensively reported. However, should targeting multiple antigens cause an increase in off-target cytotoxicity, strategies have been devised to circumvent this issue, including targeting tumor-restricted post-translational modification, as well as “AND” or “NOT” Boolean logic gates (discussed below) [67,68,69]. These strategies may be implemented to increase specificity further and reduce off-target effects.

## 3. Logic Gated CAR T Cells

### 3.1. Introduction to Logic Gates

**Figure 3:**
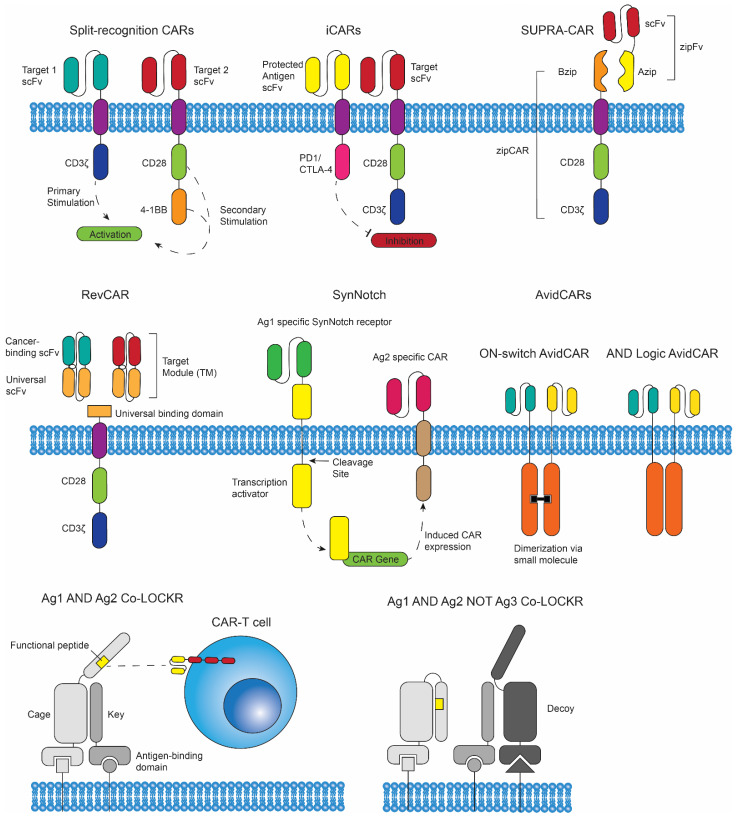
**Various applications of logic gated CAR constructs.** Split-recognition CARs separate the primary (CD3ζ) activation domains from the co-stimulatory domains (CD28/4-1BB), with each having separate scFv binding regions. Activation of both scFvs will produce a combined activation signal to the T cell. Inhibitory CARs (iCARs) use a separate inhibitory construct to prevent activation of the CAR T cell when the protected antigen scFv is bound. The inhibitory activation domain uses the signaling domains of inhibitory coreceptors (PD-1/CTLA-4) to produce an inhibitory signal when bound to the protected antigen and prevents CAR T cell activation, even when the target scFv is bound. SUPRA CARs have a zipCAR, which is composed of classic transmembrane and activation domains bound to a basic leucine zipper (Bzip). The zipFv, composed of a Azip region and the scFv, is administered separately, which binds to the Bzip of the zipCAR, allowing for activation of the SUPRA CAR. RevCARs have a classic CAR construct, but the scFv is replaced with a universal binding domain. Target modules (TMs) are administered separately, which are composed of an scFv specific for the universal binding domain combined with the cancer-binding scFv. The TMs bind to the CAR construct and activate the RevCAR. SynNotch receptors can be utilized to restrict CAR T cell activation by requiring a priming step. When the SynNotch receptor binds to its specific antigen, protease cleavage releases the intracellular transcriptional activator domain to induce the expression of a CAR specific for another antigen. AND logic gates may be applied to AvidCARs, which apply avidity effects and dimerization of signaling domains to elicit CAR T cell anti-tumor functions. ON-switch AvidCARs only induce CAR T cell activation when both antigen-binding regions bind their target antigen and when a dimerization molecule is administered. AND logic AvidCARs require both antigen-binding domains to recognize Ag1 and Ag2 to induce CAR T cell activation without the need of a dimerization molecule. Co-LOCKRs may consist of a cage protein that contains a latch domain, which sequesters a functional peptide, one or more key proteins, and a decoy protein. For AND logic gated Co-LOCKRs (Ag1 and Ag2) directing CAR T cell cytotoxicity, both the cage and key proteins must bind their cognate antigen presented on cancerous cells for colocalization and exposure of the functional peptide to occur. Once this happens, a CAR T cell with a binding domain specific to the exposed functional peptide will bind to the Co-LOCKR and direct its cytotoxic effects towards the antigen-bearing cancer cell. AND/NOT gated Co-LOCKRs (Ag1 AND Ag2 NOT Ag3) perform AND logic functions similarly when Ag1 and Ag2 are present but not Ag3. When Ag3 is present, the decoy protein localizes with and binds to the key protein and inhibits the exposure of the functional peptide.

In addition to multi-specificity in CAR constructs, Boolean logic is a recent advancement that allows for greater specificity of CAR T cells to cancer [70]. Logic terms such as AND or NOT refer to activation restrictions that limit CAR T cell activation to either activate only in the presence of two target antigens, or to activate only in the presence of the target antigen and to remain inactive when in the presence of the off-target antigen (Figure 3). AND logic gating can be applied by separating the multiple activation signals, such that one receptor contains the primary activation signal (CD3ζ) and the second construct contains the co-stimulatory domains (CD28/4-1BB). These CAR constructs can be referred to as split-recognition CAR T cells. NOT gate logic can be achieved by including inhibitory signaling in place of activation domains as the internal signaling component of the off-target CAR construct. When in the presence of the off-target antigen, inhibitory signals will prevent the activation of the CAR T cell. These CAR T cells are known as inhibitory CARs (iCARs) [71]. Both AND and NOT logic gates can be applied together in a single CAR T cell construct.

### 3.2. SUPRA CARs, RevCARs, SynNotch CAR T Cells, and AvidCARs

Another CAR modification using AND logic gating is a split universal programable (SUPRA) CAR. In this design, a zipCAR is introduced into a T cell, containing a basic leucine zipper (Bzip) binding domain combined with transmembrane and intracellular signaling domains [72]. A zipFv is also engineered, which includes an Azip domain (which binds to the Bzip region) bound to an scFv (Figure 2). This lock and key method of attaching to the binding domain has several advantages, including the ability to switch the antigen binding domain, fine-tune activation through adjustment in Azip/Bzip binding affinity, and combine multiple scFvs in one therapeutic approach. SUPRA CARs were further developed to have a separate inhibitory domain, adding NOT logic to their capabilities [73]. In mouse models for leukemia and breast cancer, SUPRA-CARs had an anti-tumor effect comparable with conventional CAR T cells; their versatility may make them a more promising option [72,73].

RevCARs are an application of AND and OR logic gating, where a CAR T cell with a universal binding domain is combined with different target modules (TMs) [74]. TMs are comprised of two scFv regions linked together, one that binds to the universal binding domain on the CAR and one that binds to a cancer antigen. This allows the RevCAR to target a variety of antigens based on the TM administered in conjunction. In vitro tests, in vivo studies in several cancerous mouse models, and studies with patient derived acute myeloid leukemia cells show potent anti-tumor effects, supporting further research of this technology [74,75].

Similar to AND logic gating strategies, synthetic notch (SynNotch) receptors, which function by inducing the expression of effector proteins upon antigen recognition, have been introduced into CAR T cells to apply an “if-then” strategy; if the SynNotch receptor recognizes its antigen, then expression of a CAR specific to another antigen is induced (Figure 3) [76,77]. SynNotch receptors were first introduced into CAR T cells by Roybal et al. in 2016 to enhance specificity. The authors demonstrated that once armed and activated, SynNotch CAR T cells would specifically eliminate dual-antigen bearing cells, while leaving single-antigen cells unharmed in vivo. Thus, CAR T cells can be armed and exhibit their directed, cytotoxic functions towards cells bearing a priming antigen and a TAA, while localizing cytotoxicity within tumors. However, off-target toxicity may still be an issue if SynNotch and CAR-specific antigens are present on healthy cells in proximity to the tumor [41]. In 2021, Choe et al. applied this SynNotch strategy to CAR T cells primed by the epidermal growth factor receptor splice variant III (EGFRvIII) and myelin oligodendrocyte glycoprotein (MOG) to treat glioblastoma [78]. Their results show that SynNotch CARs were effective at killing glioblastoma tumor cells in vivo while localizing CAR T cell priming within tumors. Interestingly, the authors reported that the SynNotch CAR T cells exhibited reduced exhaustion and improved persistence in vivo, compared to T cells bearing constitutively expressed CARs, and their results suggest that this may be the result of reduced tonic signaling.

AvidCARs, introduced by Salzer et al. in 2020, capitalizes on the fact that in many natural immune reactions, interactions of low-affinity antigen-binding domains with their ligand are amplified by avidity effects [79]. The authors engineered several generations of AvidCARs incorporating ON-switch and AND logic gate strategies. All of their AvidCAR constructs required CAR dimerization and incorporated at least two low-affinity antigen-binding domains. The authors were able to control dimerization of the CAR, and therefore its downstream effects, by the addition or removal of a dimerization molecule or by requiring dual-antigen recognition (AND logic gating) for dimerization to occur. A combination of AND logic gating and the need for a small molecule for dimerization can lead to even greater specificity and localization of CAR T cell activation and anti-tumor functions. The authors investigated the efficacy and specificity of ON-switch AvidCARs (needs dimerization molecule) and AND logic gated AvidCARs (only dual-antigen recognition needed) in vivo (Figure 3), and they report that both constructs were effective at killing specifically antigen-bearing cancer cells, while not exhibiting any cytotoxic effects without the requirements being met. Remarkably, the AND-logic AvidCAR did not kill single-antigen bearing cells, even when both antigens were present on mixed single-positive cells in close proximity.

### 3.3. Co-LOCKRs

Colocalization-dependent latching orthogonal cage-key proteins (Co-LOCKR) are protein switches that can be configured to perform AND, OR, and NOT Boolean logic operations. These proteins were developed by Lajoie et al, and they consist of a structural “cage” protein that contains a “latch” domain that sequesters a functional peptide in an inactivated conformation, a “key” protein that binds to the cage and induces a conformational change that exposes the functional peptide, and an optional second key to include NOT or OR logic to the system [80]. Co-LOCKRs may target two or three antigens on cells using targeting domains attached to the cage and key proteins. The authors were able to direct CAR T cell cytotoxicity using Co-LOCKRs (CL_C_Antigen_K_Antigen_; CL = Co-LOCKR, C = cage, K = key) specific to cells expressing both Her2 and EpCAM (CL_C_H_K_Ep_), with limited killing of single-antigen cells. In their model, the CAR contained a stabilized variant of human Bcl2 that would bind to Bim, which was encoded into the latch as a sequestered peptide. Upon binding of the cage and key proteins to their target antigens, Bim would be exposed and the Bcl2 CAR would bind to Bim and exhibit its cytotoxic effects. They reported similar success in selective cytotoxicity when targeting Her2 and EGFR (CL_C_H_K_E_) and when switching the binding domains between the cage and key proteins (CL_C_E_K_EGFR_ and CL_C_Ep_K_H_). Their results demonstrate the ability of Co-LOCKRs to direct and restrict T cell cytotoxicity to only those tumor cells expressing both antigens. They went on to test the feasibility and efficacy of combining AND/OR logic gates (CL_C_Ag_K_Ag_K_ag_), as well as AND/NOT logic gates (CL_C_Ag_K_Ag_K_D_, D= decoy, which sequesters the key protein) (Figure 3). The AND/OR Co-LOCKRs (CL_C_H_K_E_K_Ep_, CL_C_E_K_H_K_Ep_, and CL_C_Ep_K_H_K_E_) each successfully directed CAR T cell functions toward antigen bearing K562 and Raji cells, as measured by IFNγ production, proliferation, and cytotoxicity. The authors tested AND/OR logic gated Co-LOCKRs (CL_C_H_K_Ep_D_E_) and found that IFNγ production and proliferation was induced when K562/Her2/EpCAM cells were present, but not when EGFR was expressed on these cells (K562/EGFR/Her2/EpCAM). Importantly, the authors stated that the antigen targeted by the decoy protein needed to be expressed at higher levels than the alternative antigen to be targeted for the NOT logic gate to work.

## 4. CAR NK Cells

### 4.1. Natural Killer Cells in Cancer Immunity

**Figure 4:**
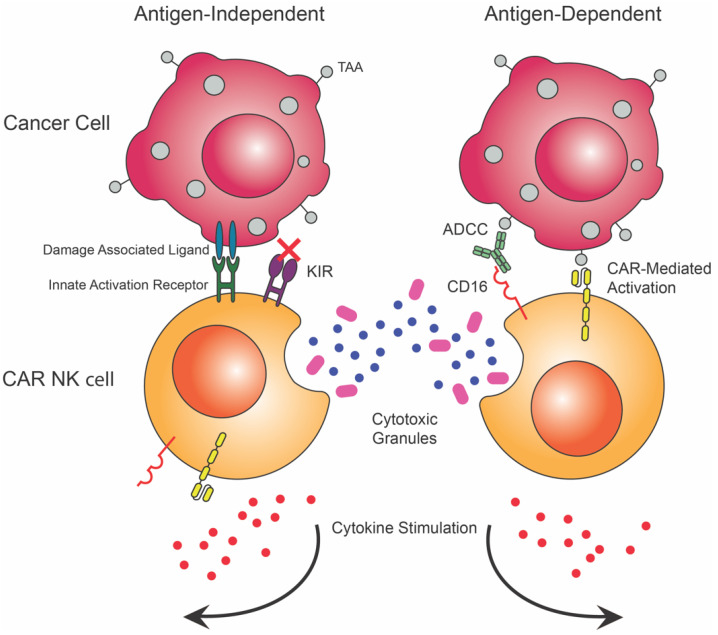
**Natural killer CAR cell antigen independent and antigen dependent functions**. Antigen-independent targeting of cancer is shown (**left**) through innate recognition. Antigen-dependent recognition is shown (**right**) through CAR recognition of TAAs. Antibody-dependent cellular cytotoxicity (ADCC) is also shown through binding of CD16 to tumor specific antibodies.

Another cell type being studied for cancer immunotherapy are NK cells. NK cells are innate cytotoxic lymphocytes that can recognize viral infection, cancers, and stressed cells. NK cells can initiate an antibody mediated cytotoxic response through their antibody Fc receptor FcγRIII (CD16) [81]. NK cells can innately recognize cancerous cells in an antigen-independent manner through HLA downregulation on the cancer, as well as through the recognition of damage patterns that may be on the surface of cancerous cells. CAR NK cells additionally target cancer in an antigen-dependent manner through the receptor stimulation allowing for bimodal action of CAR NK cells [82,83,84,85,86,87] (Figure 4).

Current CAR NK cell constructs are derived from CAR T cell constructs and frequently include the same internal signaling domains as CAR T cell constructs, such as CD3ζ, CD28 and 4-1BB. Because these internal signaling domains are tailored to T cells, there may be improvement if these domains were replaced with receptor domains more commonly associated with NK cells. Recent studies have tested the efficacy of using NK specific intracellular domains, such as DAP12 and DAP10. These CAR NK cells have been equally as effective as CARs with T cell internal signaling domains in ALL, osteosarcoma, and prostate cancer [88,89]. CAR NK cells are advantageous because adoptive cell transfers do not induce GvHD, and HLA matching is not required [90,91,92]. This allows for the potential of an off-the-shelf treatment option. While NK cells with HLA-mismatching may not induce GvHD, the effectiveness of HLA-mismatching is in discussion and may be limited by the rejection of HLA-mismatched NK cells, or in contrast, may enhance the treatment as HLA mismatched cancer cells are more readily recognized by donor NK cells [93,94]. Additionally, when compared to CAR T cells, CAR NK cells pose less of a threat in terms of CRS and neurotoxicity, making them a safer option than current CAR T cell therapies [90,92]. NK cells have also been shown to develop a subset of memory or memory-like cells that have been utilized in some cancer immunotherapy strategies and may provide an additional benefit in the creation of CAR NK cell therapies [95,96,97].

NK cells are found primarily as immature (CD56^high^, CD16^-^) or mature (CD56^low^, CD16^+^) cells throughout the blood and can be found localized in lymph nodes [98]. NK cell antigen-independent activation is regulated by a balance between activating surface receptors and repressive receptors. Killer Ig-like receptors (KIRs) present on NK cells bind to MHC and allow the NK to recognize self-cells and prevent cytotoxicity against healthy cells [99]. Binding of MHC additionally provides licensing for immature NK cells to be able to elicit a cytotoxic response [99]. Other receptors such as toll-like receptors (TLRs), can recognize damage-associated molecular patterns (DAMPs) and stress signals that provide activation signaling to the NK cell. Mature NK cells will also participate in ADCC by the binding of CD16 to the Fc region of an antibody, causing a release of perforins and granzyme B. In addition to providing an innate cytotoxic response, NK cells produce pro-inflammatory cytokines, including TNF-α and INFγ [98]. NK cells pose an interesting option for use in cancer immunotherapies, due to their innate action against cancer both through cytotoxic recognition of cancer and in their production of anti-angiogenesis pro-inflammatory cytokines [100]. Advantages of CAR NK cells can be classified by availability, safety, and efficacy as a treatment.

### 4.2. Availibility

NK cells, in contrast to T cells, can be obtained from allogeneic sources, including umbilical cord blood, human embryonic stem cells (hESCs), induced pluripotent stem cells (iPSCs), peripheral blood, and NK-92 cell lines [101]. Obtaining enough NK cells for engineering remains a challenge to CAR NK cell development, due to the unique challenges associated with each source. Peripheral blood and umbilical cord blood, although more widely available, can provide a fairly heterogenous mixture of NK cells, which diminishes the yield of fully mature NK cells [102]. NK-92 cells are derived from non-Hodgkin lymphoma, but they must be irradiated prior to use in treatment, which diminishes cell expansion. iPSCs and hESCs require more processing but produce a more homogenous mixture of cells [103,104,105]. A concern with NK cell availability is that NK cells once transplanted, tend not to expand sufficiently in vivo and require ex vivo expansion to be viable as a treatment option [106,107,108]. In addition to allogeneic sources, NK cells can be obtained from the patient and expanded ex vivo, the same as other NK cell sources.

### 4.3. Saftey

CAR NK cells appear to be a safer option for immunotherapy than CAR T cells [103]. CAR NK cells, while maintaining a similar level of cytotoxic effect, have not had CRS or neurotoxic effects to date [109]. Liu et al. in a key clinical trial found that CD19 CAR NK cells, obtained from HLA mismatched cord blood, were not associated with GvHD, CRS, or neurotoxicity, highlighting the safety of CAR NK cells as a treatment. Although NK cells participate in proinflammatory signaling, the cytokines released by NK cells are different than the release of T cells and do not elicit the same overactivation present in CRS. Additionally, NK cells in contrast to T cells can be obtained from allogeneic sources without fear of GvHD when not in a transplant setting [90,92,103,110,111,112,113].

### 4.4. Efficacy

CAR NK cells provide a bimodal method of targeting cancer cells both through antigen-dependent and independent mechanisms. This becomes much more important in heterogenous tumors that have higher diversity in cell type. The bimodal action of CAR NK cells can potentially aid in cancer escape prevention and the removal of cancer cells [114]. CAR NK cells were developed as an alternative to CAR T cell therapies to address some of the potential issues present in CAR T cell therapies. The first CAR NK clinical trials began in 2016, and currently there are 32 CAR NK cell therapies in clinical trials, with many of them aimed at targeting blood cancers through BCMA and CD19 antigen-targeting [109,115,116]. Currently, CAR NK cells are a viable option for cancer immunotherapy development but face challenges in ex vivo cell expansion, storage conditions, and optimal cellular cytotoxicity. Additionally, CAR NK cells, such as CAR T cells, face the challenges of tumor infiltration but with stimulation may be able to increase efficacy [117].

## 5. CAR Macrophages

### 5.1. Macrophages in Cancer Immunity

Macrophages are phagocytic innate immune cells that play major roles in the immune response, including (1) phagocytosing bacterial pathogens, host cells infected with intracellular pathogens, cellular debris, and cancerous cells; (2) presenting antigens to T cells; (3) recruiting and activating effector immune cells via chemokine and cytokine secretion; (4) and cellular cytotoxicity [118,119]. Macrophages are activated upon recognition of conserved molecular motifs on pathogens, also known as pathogen-associated molecular patterns (PAMPs), or DAMPs released due to cell and tissue damage via pattern recognizing receptors (PRRs) [120]. These receptors include TLRs, NOD-like receptors (NLR), C-type lectin receptors, and RIG-I-like receptors [121].

Most monocyte/macrophages originate from myeloid progenitor cells in the bone marrow, and they circulate in peripheral blood until they are attracted to sites of infection or tumors via chemokine signaling [122]. However, most tissue-resident monocytes/macrophages are established during embryonic development and self-replenish through local proliferation throughout adulthood [123,124,125]. Based on microenvironmental stimuli, such as cytokines, macrophages may polarize (or mount a distinct functional phenotype) into two polarization states (M1 and M2), and these two polarization states perform different functions during an immune response [118,126]. M1 polarized macrophages, or classically activated macrophages, are proinflammatory and promote the activation of the adaptive immune system. M1 polarization is typically generated by GM-CSF and Th1 cytokine signaling (i.e., IFN-γ or TNF-α) during a cell-mediated immune response or by recognition of lipopolysaccharide (LPS) [118,127,128]. M1 macrophages secrete proinflammatory cytokines, such as TNF-α, IL-1α/β, IL-6, IL-12, and IL-23, which generate an inflammatory environment; and they exhibit anti-microbial and anti-tumor activity [118,129]. M2 polarized macrophages are immunoregulatory, and they promote angiogenesis and tissue repair, serving as a counterbalance to the effects of M1 polarization. M2 polarization is generated by type 2 helper T (Th2) cell cytokine signaling, specifically IL-4 and IL-13 signaling via STAT6, during the humoral-mediated immune response against parasitic infections [130,131,132]. Other cytokines, such as IL-10 and IL-21 secreted by Th2 cells and TGF-β, also influence M2 polarization [131,133]. M2 macrophages secrete immunoregulatory cytokines, such as IL-10 and TGF-β, and they are capable of phagocytosing apoptotic cells and cellular debris [129]. M2 macrophages are associated with tumor survival and progression [118]. Remarkably, M2 macrophages may switch polarization states when exposed to M1 promoting stimuli, and the same is true vice versa [118].

In the TME, macrophages play opposing roles depending on their M1- or M2-like polarizations, destroying tumor cells or promoting angiogenesis and tumor growth. Tissue-resident and circulating macrophages are recruited to tumor sites via cytokines and chemokines released by tumors, such as M-CSF, GM-CSF, IL-8, CCL20, VEGF, and CCL2 [134,135,136]. As macrophages enter solid tumors, cytokines such as IL-10 and TGF-β released by tumor cells may promote the development of immunosuppressive TAMs, which promote tumor growth and metastasis, and suppress the anti-tumor activity of infiltrating T cells [133,137]. TAMs themselves also release IL-10 and TGF-β, which in turn promote the further differentiation of monocytes into TAMs [133,135,138,139]. TAMs typically have phenotypes that resemble M2 polarization states. They exhibit poor cytotoxicity against tumor cells, present antigens poorly, promote the survival and proliferation of tumor cells, and are associated with tumor metastasis [135,140,141].

Since macrophages are phenotypically plastic, recent studies have investigated macrophage-targeted immunotherapies to promote M1 macrophage polarization and reduce the frequency of TAMs within TMEs, including reprogramming TAMs to express tumoricidal phenotypes, altering the recruitment and composition of myeloid-derived cells (including monocytes/macrophages) within the TME, and enhancing the activation of monocytes/macrophages [142]. Recent studies have shown that inhibiting the CCL2-CCL2R axis, which promotes the recruitment of monocytes into tumors that potentially polarize into M2-like macrophages, reduces tumor progression and metastasis [141,143,144,145]. TLR and CD40 agonists have been investigated to enhance the activation of macrophages and dendritic cells in the TME, including FDA-approved imiquimod (TLR7), G100 (TLR4), SD-101 (TLR9), lefitolimod (TLR9), and APX-005M (CD40) [142]. Inhibiting immunosuppressive pathways is another approach to enhancing the activation and tumoricidal functions of TAMs, including the CD47-SIRPα axis and adenosine A2A receptor pathways [142]. Approaches to reprogramming immunosuppressive macrophages into inflammatory macrophages include inhibiting the functions of class I and class II histone deacetylases, inhibiting macrophage receptor with collagenous structure (MARCO) signaling, activating CD11b signaling, and inhibiting PI3Kγ signaling [142,146,147,148,149,150,151]. These approaches to enhancing macrophage anti-tumor activity and inhibiting the development of immunosuppressive macrophages provide important insight into current studies investigating the potential efficacy of CAR macrophages that maintain their inflammatory phenotype in the TME.

### 5.2. Introduction of CAR Macrophages and Recent Advances

A major obstacle to CAR T cell and CAR NK cell therapies to treat solid tumors is the difficulty of infiltration by these genetically modified immune cells, due to the stromal ECM that surrounds many solid tumors [11,25]. The immunosuppressive TME also presents a challenge to immune cells by promoting immune cell phenotypes that are immunoregulatory and even tumor-promoting. Thus, researchers have begun focusing on genetically modifying macrophages, which are naturally efficient at infiltrating solid tumors and capable of maintaining their inflammatory phenotypes. As early as 2018, researchers have been introducing CARs into macrophages with the objective of directing macrophage anti-tumor functions toward tumor cells [152].

One of the earliest CAR constructs for macrophages was reported by Morrissey et al. in 2018 [152]. The authors developed a CAR macrophage to direct phagocytic activity toward tumor cells, which they termed chimeric antigen receptors for phagocytosis (CAR-P) [152]. CAR constructs against either CD19 or CD22 with phagocytic receptor signaling domains (Megf10 or FcRγ) showed targeted phagocytosis of coated beads and trogocytosis of live CD19+ Raji cells that were dependent on the ITAM-bearing intracellular signaling domains of Megf10 and FcRγ. The majority of CAR-P^Megf10^ (78%) and CAR-P^FcRγ^ macrophages (85%) exhibited trogocytosis of Raji cells, with limited whole cell phagocytosis (2 cancer cells eaten per 100 macrophages in a 4–8hr window), which the authors attributed to insufficient interactions between the CAR-Ps and target cells. Whole cell phagocytosis was improved with the addition of anti-CD47 antibodies to block the inhibition of phagocytosis (led to a 2.5-fold increase in whole cell phagocytosis of mouse IgG2a opsonized Raji B cells) and the inclusion of phagocytic effector recruitment motifs, such as the portion of the CD19 cytoplasmic domain. The CD19 motif recruits the phagocytosis-promoting p85 subunit of phosphoinositide 3-kinase (PI3K). The addition of the recruitment motif resulted in a 3-fold increase in whole cell phagocytosis, compared to CAR-Ps without the additional motif. The efficacy of trogocytosis and whole cell phagocytosis in reducing cancer cell numbers was also investigated. The authors reported that after 44hrs of coculturing Raji cells with CAR-Ps, the number of cancer cells was significantly reduced compared to the controls. CAR-P^FcRγ^ and CAR-Ps containing the CD19 signaling motif cleared Raji cells with similar efficiency. Thus, CAR technology introduced into macrophages seemed to be a viable immunotherapy option.

Since the introduction of CAR-Ps, different strategies for engineering CAR macrophages have been investigated. In 2019, an article published by Zhang et al. reported the development of CAR macrophages that target the ECM of solid tumors by promoting the secretion of metalloproteinases (MMP) to degrade ECM and basement membrane components [153,154]. By destroying the ECM, which acts as a physical barrier in many cancer types, the infiltration of effector immune cells and anti-cancer drugs could be improved and tumoricidal activity enhanced [155]. Using HER2 specific CAR-147 macrophages containing the intracellular domain of CD147, the authors demonstrated that the CAR-147 macrophages promoted the secretion of metalloproteinases (MMP3, MMP9, MMP10, MMP11, MMP12, MMP13, and MMP14) but had no effect on macrophage phagocytosis, ROS production, or inflammatory cytokine secretion in vitro. The in vivo results did not show altered infiltration of macrophages, altered macrophage phenotype, nor a significant increase in inflammatory cytokines (IFNγ, TNFα, and IL-6) in serum. However, they did observe increased levels of IL-12 and IFNγ within the tumors, significant improvement of CD3+ T cells, and an upregulation of MMPs (MMP2, MMP3, MMP9, MMP10, MMP11, MMP12, MMP13, MMP14, and MMP15).

In 2020, another class of CAR macrophages (CAR-M) were developed by Klichinsky et al, with the goal to overcome the tumor infiltration challenges present in CAR T cell therapies by utilizing the inherent ability of macrophages to infiltrate tumors [156]. The authors showed that both anti-CD19 and anti-HER2 CAR-Ms containing CD3ζ or FcRγ signaling domains were capable of phagocytosing antigen-bearing tumor cells in an antigen-specific manner in vitro. In two mouse models of lung metastases and intraperitoneal carcinomatosis using SCOV3 cells, CAR-M treatment significantly reduced tumor burden, as measured by bioluminescence imaging. In the lung metastases model, CAR-M treatment prolonged the overall survival of the mice from 63 days (mice treated with macrophages transduced with an empty vector that did not contain the CAR construct) to 88.5 days. In the IP carcinomatosis model, about 50% of the CAR-M treated mice survived 100 days, whereas none of the non-treated mice nor untransduced macrophages (UTD) treated mice lived past 60 days. UTD macrophages did not exhibit anti-tumor activities in their in vitro or in vivo models. Unlike CAR-147s, as described by Zhang et al., Klichinsky et al. observed with single-cell RNA sequencing (scRNA) that the CAR-Ms clustered toward M1 polarization and maintained their M1 phenotypes within the TME, expressing M1-associated genes (IFIT1, ISG15 and IFITM1) and lacking the M2 marker MRC1. This is noteworthy, since the TME often exerts a dominant immunoregulatory influence on infiltrating leukocytes [155]. Additionally, scRNA revealed that surrounding cells within the TME, excluding adoptively transferred macrophages, had increased transcription of proinflammatory genes, such as HLA genes and TNF. Thus, CAR-Ms promoted an inflammatory state within the TME. Further investigation of the CAR-M’s influence on the inflammatory state of the TME revealed that CAR-Ms induced pro-inflammatory pathways in M2-polarized macrophages in vitro, induce activation and maturation markers (CD86 and MHC class II molecule) in immature human dendritic cells, and induced the recruitment of both resting and activated human T cells compared to UTD. The authors also demonstrated that CAR-Ms were capable of antigen cross-presentation to tumor-specific CD8+ T cells. Thus, CAR-M therapy has the benefits of not only overcoming the challenges of maintaining anti-tumor functions in the TME, but they enhance the anti-activity of other effector cells by manipulating the TME and presenting antigens. Since the publication of this article, which was performed by Carisma Therapeutics, an ex vivo gene-modified autologous CAR-M (CT-0508) has begun phase 1 of clinical trials as an intended cellular therapy to treat solid tumors that overexpress HER2 (NCT04660929).

**Figure 5:**
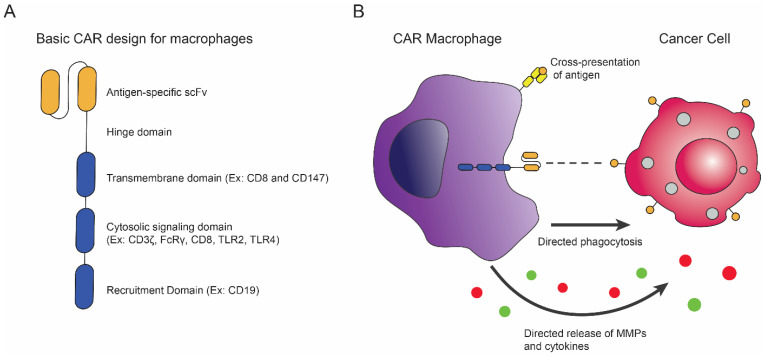
**Basic CAR design and effector functions of CAR macrophages**. (**A**) The basic CAR design for macrophages includes an antigen-specific scFv region that binds to antigen on target cells, a hinge domain, the transmembrane domain that can be derived from portions of molecules, such as CD8 and CD147 (as previously described by Morrissey et al, Klichinsky et al., and Zhang et al.), a cytosolic signaling domain that promotes phagocytosis or the release of MMPs and cytokines depending on the domain used, and an optional recruitment domain to enhance signaling (such as CD19, Morrissey et al.). (**B**) The anti-tumor functions of macrophages can be directed towards cancer cells, including phagocytosis and the release of inflammatory cytokines and metalloproteinases (MMP). Additionally, CAR-Ms can promote the activation of cytotoxic T cells by cross-presenting tumor associated or tumor specific antigens.

It is worth noting the use of different intracellular domains in the studies described above to promote specific macrophage functions to reach a desired result (i.e., phagocytosis of whole tumor cells or the secretion of MMPs) (Figure 5). Even the use of TLR intracellular signaling domains are being investigated as components of CARs (described below) [157]. Thus, the choice and optimization of which intracellular domain to direct macrophage anti-tumor activities is a very important step when developing CAR-M therapies. The choice of the viral vector used to transduce macrophages with CARs may also have an influence on the phenotype of CAR-Ms, as observed between the Zhang et al. and Klichinsky et al. studies, in which the former used a lentiviral vector and reported no phenotypic alterations and the latter used an adenovirus vector, which seemed to promote M1 polarization [155,156].

### 5.3. Alternative CAR Macrophage Designs and Therapies

Genetically manipulating macrophages can be difficult due to their proficiency at detecting and rejecting foreign genetic material [158,159]. The development of novel viral vectors and non-viral vectors have been some approaches to solving this problem [160,161,162,163]. A relatively new approach to addressing the difficulty in genetically engineering CAR macrophages from bone marrow or peripheral blood mononuclear cells (PBMC)-derived monocytes/macrophages is the development of iPSC-derived CAR macrophages, or CAR-iMacs [164]. Studies have demonstrated that PBMCs may be isolated from a host and reprogrammed to become iPSCs. These iPSCs are engineered to express a CAR and induced to differentiate into myeloid/macrophage lineages that express typical macrophage markers [164]. However, CAR-iMacs demonstrate strong M2 polarization based upon metabolism gene expression [164].

A novel class of CARs in development is the macrophage toll-like receptor chimeric antigen receptor (MOTO-CAR™), which contains a TLR intracellular signaling domain [157]. Thus far in their development and testing, RAW 264.7 murine macrophages have been electroporated to introduce the MOTO-CAR™ with an anti-mesothelin scFv region and either the TLR4 or the TLR2 intracellular signaling domains into the macrophages, and the functionality of the MOTO-CARs™ was tested in vitro by co-culturing the transfected macrophages with a mammary gland squamous carcinoma cell line (HCC-1806). The authors reported efficient tumor elimination at effector:target ratios of 1:10 (TLR2 and TLR4 MOTO-CARs), 1:5 (TLR4 MOTO-CAR), and 2:1 (TLR2 and TLR4 MOTO-CARs), compared to non-transfected macrophages (mock) controls. The authors also reported a significant increase in TNF-α secretion at 24hrs (TLR2 and TLR4 MOTO-CARs) and 48hrs (TLR4 MOTO-CAR only) of incubation with CC-1806 cells, when compared to the mock controls. Upon testing the effect of MOTO-CARs™ on tumor burden in NSG mice containing HCC-1806-derived xenografts, they observed a 46% decrease in tumor weight in TLR4 MOTO-CAR treated mice compared to mock mice. They also reported inhibited tumor growth and a 360.1% increase in tumor size (mm^3^) in TLR4 MOTO-CAR treated mice, compared to a 503% increase in tumor size in mock mice. Similar results were observed by the authors when MOTO-CARs™ derived from primary human monocytes were co-incubated with the same target cells [157].

## 6. CARs beyond Cancer

### 6.1. CARs in Autoimmune Disease

**Figure 6:**
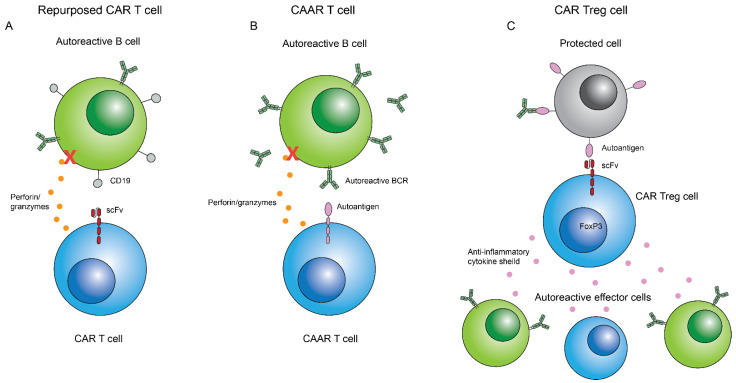
**Three approaches to treating autoimmune disease with CAR T cells**. (**A**) Repurposed CAR T cells are standard CAR T cells with scFvs generated against an epitope for an autoimmune disease. They use standard CD8 T cell killing mechanisms, including perforin and granzymes. (**B**) Chimeric autoantigen receptor (CAAR) T cells are a novel approach that use an epitope of an autoantigen as the binding domain of the CAAR T cell. This allows the CAAR T cell to bind to autoreactive B cell receptors (BCRs) and eliminate the autoreactive B cell through standard CD8 T cell mechanisms. (**C**) CAR Treg cells have an scFv against an autoantigen that has disease causing autoantibodies produced against it. The CAR Treg cell binds to this autoantigen on the cell that needs protecting and releases anti-inflammatory cytokines, producing a shield to prevent autoreactive effector T and B cells from destroying the protected cell.

The success of CAR T cells in treating cancer has led researchers to question if this concept can be applied to other diseases. Autoimmune diseases are a natural CAR application because they are often mediated by lymphocytes, and thus are more easily targeted by CAR T cells and avoid difficulties related to the TME compared to cancerous cells found within solid tumors [165]. There are three main approaches to treating autoimmune disease with CAR T cells, which are as follows: (1) repurposed standard CAR T cell constructs for specific autoimmune epitopes, (2) Chimeric autoantigen receptor (CAAR) T cells, which use an autoantigen as bait for autoreactive B cells, and (3) CAR regulatory T cells (Treg) cells, which can counteract the chronic inflammatory response of autoimmune diseases (Figure 6).

CAR T cells also have emerging promise in other infectious and chronic diseases, such as HIV and cardiac fibrosis, which are beginning to be explored [166].

### 6.2. Repurposing CAR T Cell Constructs for Autoimmune Disease

CAR T cells as used in cancer could also provide a treatment option for autoimmune disease when scFvs specific for autoantigens, which lead to disease in autoimmune diseases, are used. Systemic lupus erythematosus (SLE) is a complex autoimmune disease involving multiple organs; however, the primary effector cells are autoreactive B cells, which produce disease-causing autoantibodies [167]. These autoantibodies commonly bind to extracellular DNA and other intranuclear proteins forming immune complexes, generating systemic chronic inflammation. B cell depletion has been one approach for the treatment of SLE, since it would reduce or eliminate the production of autoantibodies. An anti-CD20 monoclonal antibody, Rituximab, was tested in clinical trials for SLE, but was only partially effective at eliminating B cell populations [168,169,170]. Kansal et al. improved this method of SLE treatment by repurposing second generation CD19 CAR T cells, originally used for lymphomas, to deplete the entire B cell population in two different accepted SLE mouse models [171]. IgG and IgM levels were successfully depleted after CD19 CAR T cell treatment in both mouse models. Furthermore, CD19 CAR T cell treatment improved SLE disease progression and survival rates of mice compared with the controls. CD19 CAR T cell therapy for the treatment of SLE is currently under phase 1 of clinical trials (NCT03030976, NCT05030779) [171]. One limitation to CD19 CAR T cells in treatment of SLE is that patients will likely experience humoral immunosuppression, because all B cell populations and resulting antibodies are depleted [40]. As in cancer treatment with CD19 CAR T cells, patients can receive transferred humoral immunity to counteract the treatment induced immunosuppression [172]. This repurposing of CD19 CAR T cells beyond their use for cancer may prove an effective and long-lasting therapy for patients with moderate to severe SLE and could be an option for other diseases where complete B cell depletion would provide relief.

CAR T cell therapy is also being used to treat type 1 diabetes, an autoimmune disease where multiple types of immune cells are involved in the destruction of insulin-producing beta cells. Fishman et al. created a muti-specific CAR T cell with a receptor targeting different epitopes on the MHC 1 complexes of autoreactive CD8 T cells that are believed to be central to the development of type 1 diabetes [173,174]. Their multi-specific CAR T cell with a peptide/β2m/CD3-ζ receptor was effective at eliminating insulin-reactive T cells in vitro and when injected into NOD mice [173]. Another approach to treating diabetes with CAR T cells was proposed by Zhang et al., where a monoclonal antibody (mab287), that recognizes a common MHC-II/insulin peptide complex, I-Ag7-B:9–23, was engineered into a CAR T cell [175]. mab287 is shown to block recognition of insulin by T cells and delay development of type 1 diabetes by at least 10 weeks [176]. mab287 CAR T cells eliminated APCs presenting the I-Ag7-B:9–23 complex relatively well in vitro and when injected into NOD mice, they also delayed the development of diabetes in NOD mice for the first 30 weeks after injection [175].

CAR T cell therapies using multi-specific CARs or scFvs engineered against autoantigens could be potential options for treating other autoimmune diseases by targeting a specific subset of autoreactive cells. The difficulty lies in the discovery of conserved epitopes in autoimmune diseases that can serve as effective targets for CAR T cell therapies.

### 6.3. CAAR T Cells for Antibody Mediated Autoimmune Disease

Chimeric autoantigen receptor (CAAR) T cells are another variation in CAR T cells targeting autoreactive B cells in autoimmune disorders; however, instead of eliminating all B cells, they specifically target autoreactive B cells. CAAR T cell constructs are a novel modification to the traditional CAR T cell construct, which replace the scFv with an antigen specific for autoantibodies produced against it [166]. This method depends on the fact that B cells produce both antibodies and B cell receptors (BCRs) specific for the same antigens [177]. Thus, autoreactive B cells with autoantigen-specific BCRs are bound to the CAAR T cell expressing the autoantigen, and the B cell can be killed through traditional CD8 T cell cytotoxicity.

This idea was first used by Ellebrecht et al. in a CAAR T cell treatment for pemphigus vulgaris (PV). PV is an autoimmune disease where autoantibodies against Dsg3, a keratinocyte adhesion protein, cause blistering and inflammation [178]. CD20 targeted B cell depletion has given short remission to PV patients in the past, but relapse occurs due to incomplete B cell depletion and the return of anti-Dsg3 autoantibodies. Ellebrecht et al. hypothesized that Dsg3-specific CAAR T cells could eliminate Dsg3-specific B cells, thus preventing relapse and providing a cure to PV [179]. A panel of Dsg3 CAARs were developed with varying epitopes of the extracellular Dsg3 domain. The authors observed that Dsg3-CAAR expressing T cells exhibited successful cytotoxic effects in vitro against an engineered Dsg3 B cell line [179]. Consideration was taken as to whether soluble anti-Dsg3 antibodies would inhibit the CAAR T cell function; however, in the presence of PV serum, the soluble antibodies did not have an inhibitory effect on the CAAR cytotoxicity and may even have a beneficial effect in preventing exhaustion [179]. In a PV hybridoma mouse model, Dsg3 CAAR T cells eliminated Dsg3 B cells in vivo and reduced blistering on the mice [179]. Importantly, no off-target effects were recorded, most notably against healthy B cells [179]. Preliminary data from PV patient blood samples quantifying dosage pharmacology were published, qualifying it for progression to phase 1 of clinical trials (NCT04422912) [180]. Cabaletta Bio® is also currently performing pre-clinical investigations of CAAR efficacy in other autoimmune diseases, including myasthenia gravis and PLA2R-associated membranous nephropathy [181].

Recently, CAAR technology was repurposed to treat a blood disorder (hemophilia A) rather than an autoimmune disease by Parvathaneni et al. Hemophilia A is a hereditary bleeding disorder caused by a lack of coagulation factor FVIII in serum and is commonly treated by replacement FVIII therapy [182]. In up to 30% of patients, a drug-antibody response is generated where anti-FVIII neutralizing antibodies are formed, rendering the FVIII replacement therapy ineffective [183]. Parvathaneni et al. engineered CD8 T cells with B-cell antibody-targeting receptors (BARs), analogous to CAARs [184]. FVIII BAR T cells eliminated anti-FVIII autoreactive B cells, preventing formation of neutralizing antibodies against FVIII in engineered B cell lines and mouse models of hemophilia A, even with FVIII antibodies already present [184].

CAAR T cell therapy has the potential to treat many autoantibody-mediated autoimmune diseases, particularly organ specific diseases with one or few types of disease-causing autoantibodies [185]. Autoimmune diseases with autoantibodies against one specific autoantigen are more applicable, because one CAAR T cell could eliminate all the autoreactive B cells, providing a cure for the autoimmune disease. The precision of CAAR T cells in eliminating only disease-causing B cells reduces off-target effects and gives CAAR T cell therapy an advantage as a potential therapy to cure various autoimmune diseases.

### 6.4. CAR Treg Cells

CAR Tregs are another modification to CAR technology that are currently being investigated for the treatment of autoimmune disease. Treg cells are key to maintaining the balance in an immune response via the secretion of anti-inflammatory cytokines, and are differentiated primarily through their expression of the FoxP3 gene [186]. In many autoimmune disorders, low levels of Treg cells are present, contributing to the autoreactivity and chronic inflammation [186]. CAR Treg cells function differently compared to CAR T cells. Instead of killing target cells, CAR Treg cells release anti-inflammatory cytokines upon antigen recognition and act as a protective shield, preventing further autoreactivity [187]. One challenge to this therapy is the ability of Treg cells to switch to inflammatory phenotypes, which would exacerbate autoreactivity instead of preventing it, although researchers are aware of and are addressing this possibility by inducing FoxP3 in different ways [188,189,190].

Currently, no CAR Treg studies have progressed to human clinical trials; however, mixed results have been published in in vitro and animal studies for a variety of autoimmune diseases [187]. The first use of CAR Tregs was by Elinav et al. in a colitis mouse model with a CAR Treg specific for 2,4,6-trinitrophenol. The anti-2,4,6-trinitrophenol CAR Treg was effective at reducing colitis symptoms in mice, without reverting to inflammatory phenotypes [191]. Blat et al. also studied CAR Treg treatment in colitis mouse models, with CAR Treg constructs specific for carcinoembryonic antigen (CEA). The authors found in two colitis mouse models that the CEA CAR Tregs accumulated properly in the colon, decreased the severity of colitis, and decreased the development of subsequent colorectal cancer [192]. Recently, CAR Tregs were developed to treat an experimental autoimmune encephalomyelitis (EAE) mouse model of multiple sclerosis (MS), with an scFv targeting the myelin oligodendrocyte glycoprotein (MOG) protein, a key protein that is attacked, leading to myelin degradation. The CAR construct included the FoxP3 gene to induce the Treg phenotype and reduce switching to inflammatory phenotypes [193]. Although CAR Tregs were successful in the model for MS, a similar study of anti-insulin CAR Tregs in NOD mice was ineffective at reducing or preventing type 1 diabetes, potentially due to the multiple forms of insulin present in the body [194]. Notably, the anti-insulin CAR Tregs were only present in the mice for approximately 4 months, which is shorter than several other CAR Treg studies [194]. Finally, CAR Tregs were studied for the treatment of Hemophilia A by targeting FVIII, similarly to the study by Parvathaneni et al. [195]. The FVIII BAR Tregs were effective at inducing a more tolerogenic response by suppressing T cell and memory B cell responses; however, the BAR/CAAR CD8 T cells developed for hemophilia A were more effective at completely preventing the formation of neutralizing FVIII antibodies [195,196]. With further investigation, CAR Tregs have potential for the treatment of other autoimmune diseases and chronic inflammatory diseases.

### 6.5. CAR Use in Other Disease

CAR T cells have been attempted for use in a variety of other diseases, including viral diseases, cardiac fibrosis, and allergy/asthma. HIV application of CAR T cells was one of the first clinical trials in the 1990s, and it has been studied further since then [197,198,199]. Varying CAR strategies (from using CD4 as the binding domain to scFvs with similar structures to broadly neutralizing antibodies and combining the two) have been explored and been involved in clinical trials, but none have had clinical significance thus far [200,201]. Although HIV CAR T cells are shown to be safe and partially effective, several challenges remain, including preventing HIV infection of CAR T cells, the high mutation rate of HIV and the HIV low antigen burden, which would make targeting HIV infected cells more difficult (extensively reviewed by Mazzi et al.) [166,202]. CAR NK cells developed against SARS-CoV-2 have shown efficacy at eliminating infected cells in vitro, and are now under phase 1 of clinical trials (NCT04324996) [203]. CARs have been developed to target the antigens of other viruses (including the hepatitis B virus, hepatitis C virus, human cytomegalovirus, Epstein–Barr virus, and influenza A virus), addressing infection and related cancers [204,205,206,207,208,209,210]. These developments have had varying success in vitro and in animal models but are not yet clinically relevant.

CARs specific for non-viral pathogens are also currently under development. CAR T cells using a binding domain based on PRR Dectin-1 have been engineered to fight opportunistic *Aspergillus* fungal infections and were effective in vitro [211]. However, Dectin-1 can bind to other ligands besides β-glucans, the ligand targeted on *Aspergillus*, and their lack of specificity could make clinical application more dangerous [212]. Cardiac and liver fibrosis are currently being studied for CAR T cell therapy after the discovery of a unique endogenous marker for cardiac fibroblasts. Although, initial promising results have been difficult to replicate [213,214]. Finally, allergy and asthma are logical applications of CAR technology because these conditions are mediated in part by lymphocytes [215]. In a recent study by Ward et al., anti-IgE CAR T cells were developed to attack cells with membrane bound IgE to reduce severe allergy and asthma, but soluble IgE in high concentrations rendered the CAR T cells less effective [216]. CAR Treg based therapies for allergic asthma show some promise, but are still in the developmental stage [217]. Although CARs have potential in treating viral and other diseases, possible CRS in connection with vital organs makes them risky for patients. Specific mechanisms and pathogenesis of each disease need to be carefully considered when CARs are developed to ensure safety is properly addressed.

## 7. Conclusions

The initial success of CAR T cells in treating leukemias and lymphomas is an exciting advancement in cancer treatment that could represent the future of cancer immunotherapies. Modifications to CAR technology may provide solutions to some of the initial difficulties that CAR T cells present, especially in their application against solid tumors. CAR NK cells and CAR macrophages utilize the innate tumor recognition and infiltration capabilities of these cell types to enhance the tumor elimination properties of CARs. CAR technology also shows promise for use in other diseases besides cancer. CAAR T cells and CAR Tregs could provide treatments for autoimmune diseases by eliminating autoreactive B cells and creating an anti-inflammatory environment. Although CAR technology has wide-reaching applications, careful considerations must be taken to determine the CAR option that may be the most effective as a treatment option for that cancer or disease (Table 1). Currently, CAR T cell treatment is not the primary treatment for most cancers, and further research and development are needed to make CAR therapy safe, effective, and more widely available. Thus, the scientific community has only begun to implement the potential of CAR technologies.

## Figures and Tables

**Table 1:** List of CAR construct types with a summary of their general characteristics.

**CAR Construct**	**Mechanism**	**Benefits**	**Drawbacks**	**FDA-approved** **or Highest** **Clinical trial**
CAR T cell	CAR construct composed of extracellular domain (scFv, TCRv, Nb) with intracellular signaling domain varieties	Have been proven effective as a treatment option for B cell lymphomas Develop a memory and have a long-lasting response	Potential for CRS and Neurotoxicity MHC/HLA matching is required for donor sources Potential for off-target cytotoxicity	Six FDA-approved treatments
Multi-specific CAR T cell	Binds two or more antigens simultaneously using bicistronic CARs and CARs that utilize tandem bound scFv regions	Prevents antigen loss and potentially relapse in patients.	Potential for CRS and neurotoxicity MHC/HLA matching is required for donor sources Potential off-target cytotoxicity	Phase II
CAR NK cell	Antigen-independent and antigen-dependent modes of targeting cancer	No CRS or neurotoxicity Potential for off-the-shelf treatment Multiple donor sources available, including iPSCs, hESCs, peripheral blood, cord blood	Requires ex-vivo clonal expansion Not as potent of a response as CAR T cells Less memory than T cells.	Phase II
CAR macrophage	Directed phagocytosis or secretion of metalloproteinases to kill tumor cells or alter TME, respectively	Natural infiltration of macrophages M1 polarization promotes inflammatory TME	Difficult to genetically engineer macrophages	Phase I
CAAR T cell	Binding domain is an autoantigen that binds to autoreactive BCRs and eliminates the autoreactive B cell through standard CD8 T cell killing mechanisms	Specifically eliminates autoreactive B cells Creates memory response to prevent relapse	Most effective for autoimmune diseases with one disease causing autoantigen Potential for CRS and organ specific damage	Phase I
CAR Treg cell	The scFv binds to an antigen on a protected cell, and when bound, it signals the release of anti-inflammatory cytokines to reduce inflammation	Induces an anti-inflammatory environment with antigen specificity	Can switch to pro-inflammatory phenotypes Efficacy can be inconsistent	Preclinical

## Data Availability

Not applicable.
